# Di-*tert*-butyl *N*-[2,6-bis­(methoxy­meth­oxy)phen­yl]imino­diacetate

**DOI:** 10.1107/S1600536809009246

**Published:** 2009-03-25

**Authors:** Ben Capuano, Ian T. Crosby, Craig M. Forsyth, Yelena Khakham, David T. Manallack

**Affiliations:** a Medicinal Chemistry and Drug Action, Monash Institute of Pharmaceutical Sciences, Monash University (Parkville Campus), 381 Royal Park Parade, Parkville, Victoria 3052, Australia; b School of Chemistry, Monash University, Clayton, Victoria 3800, Australia

## Abstract

The title mol­ecule, C_20_H_31_NO_8_, has pseudo-*C*2 symmetry about the C—N bond, with the bis­(*tert*-butoxy­carbon­yl)amino group twisted from the benzene ring plane by *ca* 60° and the bulky *tert*-butoxy­carbonyl (Boc) groups are orientated away from the substituted aniline group. As part of an anti­bacterial drug discovery programme furnishing analogues of platensimycin, we unexpectedly synthesized the bis-Boc-protected aniline.

## Related literature

For the synthesis, see: Nicolaou *et al.* (2006 ▶)Khakham (2007 ▶). For related structures, see: Marino *et al.* (2002 ▶); Macleod *et al.* (2003 ▶). For the protection of amino groups in synthesis, see: ; Kshirsagar (2008 ▶).
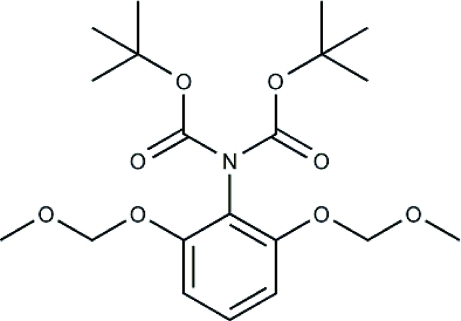


## Experimental

### 

#### Crystal data

C_20_H_31_NO_8_
*M* *_r_* = 413.46
Monoclinic, 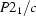

*a* = 11.2544 (3) Å
*b* = 19.6759 (6) Å
*c* = 9.8325 (3) Å
β = 93.207 (1)°
*V* = 2173.90 (11) Å^3^
*Z* = 4
Mo *K*α radiation
μ = 0.10 mm^−1^
*T* = 123 K
0.25 × 0.25 × 0.25 mm

#### Data collection

Bruker X8 APEX CCD diffractometer
Absorption correction: multi-scan (*SADABS*; Sheldrick, 1997 ▶) *T*_min_ = 0.95, *T*_max_ = 0.97
15504 measured reflections
4207 independent reflections
3714 reflections with *I* > 2σ(*I*)
*R*_int_ = 0.026

#### Refinement

*R*[*F* ^2^ > 2σ(*F* ^2^)] = 0.037
*wR*(*F* ^2^) = 0.095
*S* = 1.05
4207 reflections
264 parameters
H-atom parameters constrained
Δρ_max_ = 0.25 e Å^−3^
Δρ_min_ = −0.22 e Å^−3^

### 

Data collection: *APEX2* (Bruker, 2005 ▶); cell refinement: *APEX2* and *SAINT* (Bruker, 2005 ▶); data reduction: *SAINT*; program(s) used to solve structure: *SHELXS97* (Sheldrick, 2008 ▶); program(s) used to refine structure: *SHELXL97* (Sheldrick, 2008 ▶); molecular graphics: *X-SEED* (Barbour, 2001 ▶); software used to prepare material for publication: *CIFTAB* (Sheldrick, 1997 ▶).

## Supplementary Material

Crystal structure: contains datablocks global, I. DOI: 10.1107/S1600536809009246/pv2145sup1.cif

Structure factors: contains datablocks I. DOI: 10.1107/S1600536809009246/pv2145Isup2.hkl

Additional supplementary materials:  crystallographic information; 3D view; checkCIF report

## Figures and Tables

**Fig. 1.**
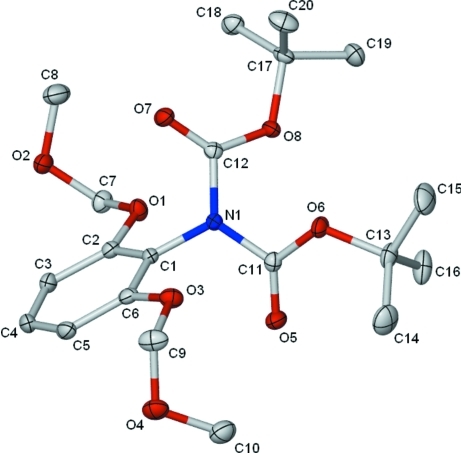
Molecular diagram of the title compound. Displacement ellipsoids are drawn at the 50% probability level.

## supplementary crystallographic information

### Comment

Protection of amino functionalities utilizing bulky *tert*-butylcarboxy groups is a common synthetic strategy in drug dicovery research programmes (Kshirsagar, 2008). Typically, mono-substituted derivatives are formed from reactions of anilines and di-*tert*-butyldicarbonate, with di-substitution generally inhibited by the poorer nucleophilic character of the intermediate secondary carbamate. In the current example, reaction of 2,6-bis(methoxymethoxy)aniline with the di-*tert*-butyldicarbonate gave 2-[bis(*tert*-butoxycarbonyl)amino]-1,3-bis(methoxymethoxy)benzene (I) in good yield. Surprisingly NMR spectra showed no evidence of restricted rotation of the *tert*-butoxycarbonyl groups in solution. The solid state structure showed a pseudo *C*2 symmetric molecule with the two methoxymethoxy arms of the benzene nucleus forming an S-shaped configuration. The bis(*tert*-butoxycarbonyl)amino fragment is twisted from the aromatic ring plane with the torsion angles C2—C1—N1—C12 57.7 (2) °; C6—C1—N1—C11 59.7 (2) ° smaller than for an analogous bis Boc aniline di-*tert*-butyl(2-(2-((4-methylphenyl)thio)-5-oxo-3-(3-oxohexyl) tetrahydrofuran-3-yl)phenyl)imidocarbonate (II) (73.6 °, 93.3 °) Marino *et al.* 2002), but larger than those observed for mono-protected anilines (*e.g.* 36.3 (3) ° in 2-(2'-*N*-*tert*-butoxycarbonyl)phenyl-1,3-dithiane (Macleod *et al.* 2003). The bulky *tert*-butoxycarbonyl groups are orientated away from the aniline group in I and II in contrast to the only other bis(*tert*-butoxycarbonyl)aniline structure, 2-(2'(*N*,*N*'-bis(*tert*-butoxycarbonyl)amino)phenyl-1,3-dithiane, in which the carbonyl groups point away from the aromatic ring (Macleod *et al.* 2003). No significant interactions between molecules of the title compound were observed (closest contact O7···H7a(#1) 2.474 Å, #1 *x*, 1/2 - *y*, 1/2 + *z*) and the observed configuration presumably derives from the steric repulsion between the *ortho* methoxymethoxy substitutents of the analine and the bulky *tert*-butoxycarbonyl groups.

### Experimental

The title compound (I) was synthesized from 2,6-bis(methoxymethoxy)aniline (Nicolaou *et al.*, 2006) and commercially available di-*tert*-butyldicarbonate in the presence of a catalytic amount of 4-(dimethylamino)pyridine (DMAP), using tetrahydrofuran as solvent (Khakham, 2007). To a 50 ml round bottom flask was added 2,6-di(methoxymethoxy)aniline (0.820 g, 3.85 mmol) and di-*tert*-butyldicarbonate (2.51 g, 11.5 mmol) and 4-(dimethylamino)pyridine (0.120 g, 0.982 mmol) and tetrahydrofuran (15 ml). The reaction mixture was heated at reflux with stirring for 24 h, cooled then evaporated to dryness. The resulting residue was purified by flash chromatography (1:4 ethyl acetate/hexane) and the major fractions were combined then evaporated to dryness. The title compound was recrystallized from dichloromethane-hexane as colorless needles (549 mg, 46%) suitable for X-ray diffraction. Mp 366–367 K.

### Refinement

All H atoms for the primary molecules were initially located in the difference Fourier map but were placed in geometrically idealized positions and constrained to ride on their parent atoms with phenyl, methyl and methylene C—H distances 0.95, 0.98 and 0.99 Å, respectively and *U*_iso_(H) = 1.5 and 1.2 times *U*_eq_(C) for methyl and non-methyl H-atoms, respectively.

### Crystal data

**Table:**

C_20_H_31_NO_8_	*F*(000) = 888
*M**_r_* = 413.46	*D*_x_ = 1.263 Mg m^−^^3^
Monoclinic, *P*2_1_/*c*	Mo *K*α radiation, λ = 0.71073 Å
Hall symbol: -P 2ybc	Cell parameters from 15504 reflections
*a* = 11.2544 (3) Å	θ = 2.1–26.0°
*b* = 19.6759 (6) Å	µ = 0.10 mm^−^^1^
*c* = 9.8325 (3) Å	*T* = 123 K
β = 93.207 (1)°	Prism, colourless
*V* = 2173.90 (11) Å^3^	0.25 × 0.25 × 0.25 mm
*Z* = 4	

### Data collection

**Table:**

Bruker X8 APEX CCD diffractometer	4207 independent reflections
Radiation source: fine-focus sealed tube	3714 reflections with *I* > 2σ(*I*)
graphite	*R*_int_ = 0.026
Thin–slice φ and ω scans	θ_max_ = 26.0°, θ_min_ = 2.1°
Absorption correction: multi-scan (*SADABS*; Sheldrick, 1997)	*h* = −13→11
*T*_min_ = 0.95, *T*_max_ = 0.97	*k* = −24→24
15504 measured reflections	*l* = −12→12

### Refinement

**Table:**

Refinement on *F*^2^	Primary atom site location: structure-invariant direct methods
Least-squares matrix: full	Secondary atom site location: difference Fourier map
*R*[*F*^2^ > 2σ(*F*^2^)] = 0.037	Hydrogen site location: inferred from neighbouring sites
*wR*(*F*^2^) = 0.095	H-atom parameters constrained
*S* = 1.05	*w* = 1/[σ^2^(*F*_o_^2^) + (0.0455*P*)^2^ + 0.7413*P*] where *P* = (*F*_o_^2^ + 2*F*_c_^2^)/3
4207 reflections	(Δ/σ)_max_ < 0.001
264 parameters	Δρ_max_ = 0.25 e Å^−^^3^
0 restraints	Δρ_min_ = −0.22 e Å^−^^3^

### Special details

**Table:**

Experimental. ^1^H NMR (CDCl_3_) δ 1.45(18*H*, s, *tert*-Bu), 3.51 (6*H*, s, OCH_3_), 5.21 (4*H*, s, OCH_2_O), 6.85 (2*H*, d, *J* = 8 Hz, H3 H5), 7.21 (1*H*, t, *J* = 8 Hz, H4). ^13^C NMR (CDCl_3_) δ 27.9 (CH_3_), 56.0 (CH_3_), 82.0 (CH_2_), 95.0 (CH_2_), 108.7 (CH), 128.7 (CH), 151.5 (C_q_), 162.0 (C_q_). ESI MS (20 V) *m/z* 844 ([2*M*+NH_4_]^+^, 25%), 414 ([*M*+H]^+^, 26%), 358 (35%), 302 (100%), 258 (40%), 182 (39%).
Geometry. All e.s.d.'s (except the e.s.d. in the dihedral angle between two l.s. planes) are estimated using the full covariance matrix. The cell e.s.d.'s are taken into account individually in the estimation of e.s.d.'s in distances, angles and torsion angles; correlations between e.s.d.'s in cell parameters are only used when they are defined by crystal symmetry. An approximate (isotropic) treatment of cell e.s.d.'s is used for estimating e.s.d.'s involving l.s. planes.
Refinement. Refinement of *F*^2^ against ALL reflections. The weighted *R*-factor *wR* and goodness of fit *S* are based on *F*^2^, conventional *R*-factors *R* are based on *F*, with *F* set to zero for negative *F*^2^. The threshold expression of *F*^2^ > σ(*F*^2^) is used only for calculating *R*-factors(gt) *etc*. and is not relevant to the choice of reflections for refinement. *R*-factors based on *F*^2^ are statistically about twice as large as those based on *F*, and *R*- factors based on ALL data will be even larger.

### Fractional atomic coordinates and isotropic or equivalent isotropic displacement parameters (Å^2^)

**Table:**

	*x*	*y*	*z*	*U*_iso_*/*U*_eq_	
O1	0.01820 (8)	0.15220 (4)	0.49516 (9)	0.0208 (2)	
O2	−0.17789 (8)	0.19162 (5)	0.45496 (10)	0.0239 (2)	
O3	0.20418 (8)	0.02812 (4)	0.84955 (9)	0.0222 (2)	
O4	0.22082 (9)	−0.08991 (5)	0.87902 (10)	0.0275 (2)	
O5	0.32175 (8)	0.04924 (4)	0.57699 (9)	0.0226 (2)	
O6	0.40454 (8)	0.13528 (5)	0.70366 (9)	0.0215 (2)	
O7	0.10556 (8)	0.22153 (4)	0.75269 (9)	0.0209 (2)	
O8	0.26357 (8)	0.23752 (4)	0.62311 (9)	0.0189 (2)	
N1	0.20550 (9)	0.13091 (5)	0.66923 (10)	0.0170 (2)	
C1	0.10389 (11)	0.08748 (6)	0.67245 (12)	0.0169 (3)	
C2	0.00578 (11)	0.09870 (6)	0.58212 (12)	0.0183 (3)	
C3	−0.09338 (12)	0.05666 (6)	0.58451 (14)	0.0224 (3)	
H3	−0.1612	0.0646	0.5247	0.027*	
C4	−0.09125 (12)	0.00289 (7)	0.67605 (14)	0.0245 (3)	
H4	−0.1581	−0.0266	0.6766	0.029*	
C5	0.00498 (12)	−0.00910 (6)	0.76637 (14)	0.0228 (3)	
H5	0.0042	−0.0461	0.8283	0.027*	
C6	0.10309 (11)	0.03395 (6)	0.76507 (12)	0.0188 (3)	
C7	−0.07663 (12)	0.16675 (7)	0.39715 (13)	0.0223 (3)	
H7A	−0.0489	0.2006	0.3315	0.027*	
H7B	−0.0976	0.1247	0.3459	0.027*	
C8	−0.15998 (14)	0.25694 (7)	0.51580 (17)	0.0344 (4)	
H8A	−0.1033	0.2531	0.5948	0.052*	
H8B	−0.2360	0.2744	0.5452	0.052*	
H8C	−0.1284	0.2882	0.4492	0.052*	
C9	0.21192 (14)	−0.02718 (7)	0.94256 (13)	0.0283 (3)	
H9A	0.2824	−0.0206	1.0061	0.034*	
H9B	0.1405	−0.0271	0.9969	0.034*	
C10	0.32667 (13)	−0.09618 (8)	0.80655 (16)	0.0331 (3)	
H10A	0.3254	−0.0626	0.7328	0.050*	
H10B	0.3310	−0.1420	0.7681	0.050*	
H10C	0.3962	−0.0882	0.8690	0.050*	
C11	0.31523 (11)	0.10078 (6)	0.64174 (12)	0.0174 (3)	
C12	0.18617 (11)	0.20088 (6)	0.68888 (11)	0.0159 (2)	
C13	0.52977 (12)	0.11794 (7)	0.67929 (14)	0.0246 (3)	
C14	0.55807 (14)	0.04678 (8)	0.73059 (17)	0.0376 (4)	
H14A	0.5129	0.0136	0.6741	0.056*	
H14B	0.5362	0.0427	0.8253	0.056*	
H14C	0.6434	0.0380	0.7255	0.056*	
C15	0.59817 (13)	0.17074 (9)	0.76495 (17)	0.0393 (4)	
H15A	0.5830	0.1640	0.8612	0.059*	
H15B	0.5721	0.2163	0.7365	0.059*	
H15C	0.6835	0.1659	0.7524	0.059*	
C16	0.55154 (13)	0.12683 (9)	0.53009 (15)	0.0351 (4)	
H16A	0.5067	0.0923	0.4767	0.053*	
H16B	0.6367	0.1217	0.5165	0.053*	
H16C	0.5253	0.1722	0.5003	0.053*	
C17	0.26643 (12)	0.31241 (6)	0.63900 (13)	0.0200 (3)	
C18	0.15042 (12)	0.34337 (7)	0.58381 (13)	0.0238 (3)	
H18A	0.1360	0.3304	0.4881	0.036*	
H18B	0.1551	0.3930	0.5910	0.036*	
H18C	0.0851	0.3267	0.6366	0.036*	
C19	0.36707 (13)	0.33176 (7)	0.54976 (16)	0.0295 (3)	
H19A	0.3453	0.3197	0.4549	0.044*	
H19B	0.4395	0.3073	0.5804	0.044*	
H19C	0.3813	0.3808	0.5563	0.044*	
C20	0.29631 (14)	0.33007 (7)	0.78745 (14)	0.0310 (3)	
H20A	0.3721	0.3088	0.8174	0.047*	
H20B	0.2332	0.3133	0.8434	0.047*	
H20C	0.3029	0.3795	0.7973	0.047*	

### Atomic displacement parameters (Å^2^)

**Table:**

	*U*^11^	*U*^22^	*U*^33^	*U*^12^	*U*^13^	*U*^23^
O1	0.0161 (5)	0.0216 (5)	0.0245 (5)	−0.0008 (4)	−0.0005 (4)	0.0046 (4)
O2	0.0167 (5)	0.0224 (5)	0.0325 (5)	0.0018 (4)	0.0003 (4)	−0.0031 (4)
O3	0.0259 (5)	0.0183 (5)	0.0222 (4)	0.0018 (4)	−0.0017 (4)	0.0016 (3)
O4	0.0336 (6)	0.0189 (5)	0.0300 (5)	0.0040 (4)	0.0022 (4)	0.0032 (4)
O5	0.0207 (5)	0.0203 (5)	0.0269 (5)	0.0022 (4)	0.0023 (4)	−0.0058 (4)
O6	0.0128 (5)	0.0250 (5)	0.0265 (5)	0.0006 (4)	−0.0003 (4)	−0.0060 (4)
O7	0.0225 (5)	0.0181 (4)	0.0227 (4)	0.0014 (4)	0.0074 (4)	−0.0008 (3)
O8	0.0200 (5)	0.0143 (4)	0.0227 (4)	−0.0020 (3)	0.0056 (4)	−0.0010 (3)
N1	0.0135 (5)	0.0146 (5)	0.0230 (5)	0.0002 (4)	0.0017 (4)	−0.0006 (4)
C1	0.0150 (6)	0.0138 (6)	0.0222 (6)	−0.0001 (5)	0.0047 (5)	−0.0026 (5)
C2	0.0176 (7)	0.0157 (6)	0.0219 (6)	0.0022 (5)	0.0050 (5)	−0.0013 (5)
C3	0.0150 (7)	0.0208 (6)	0.0314 (7)	0.0007 (5)	0.0024 (5)	−0.0028 (5)
C4	0.0177 (7)	0.0174 (6)	0.0394 (8)	−0.0031 (5)	0.0102 (6)	−0.0032 (5)
C5	0.0251 (7)	0.0142 (6)	0.0302 (7)	0.0006 (5)	0.0114 (6)	0.0019 (5)
C6	0.0203 (7)	0.0159 (6)	0.0205 (6)	0.0038 (5)	0.0042 (5)	−0.0032 (5)
C7	0.0195 (7)	0.0260 (7)	0.0210 (6)	0.0024 (5)	−0.0016 (5)	−0.0002 (5)
C8	0.0310 (9)	0.0256 (7)	0.0462 (9)	0.0045 (6)	−0.0015 (7)	−0.0094 (6)
C9	0.0428 (9)	0.0222 (7)	0.0197 (6)	0.0051 (6)	0.0017 (6)	0.0033 (5)
C10	0.0296 (8)	0.0281 (8)	0.0415 (8)	0.0070 (6)	0.0001 (6)	−0.0030 (6)
C11	0.0158 (7)	0.0188 (6)	0.0175 (6)	0.0001 (5)	0.0020 (5)	0.0025 (5)
C12	0.0170 (6)	0.0156 (6)	0.0149 (5)	−0.0004 (5)	−0.0011 (5)	−0.0001 (4)
C13	0.0123 (7)	0.0344 (8)	0.0269 (7)	0.0022 (5)	0.0004 (5)	−0.0017 (6)
C14	0.0230 (8)	0.0432 (9)	0.0461 (9)	0.0101 (7)	−0.0037 (7)	0.0073 (7)
C15	0.0194 (8)	0.0537 (10)	0.0445 (9)	−0.0062 (7)	−0.0029 (7)	−0.0104 (8)
C16	0.0192 (8)	0.0569 (10)	0.0295 (8)	−0.0030 (7)	0.0037 (6)	0.0026 (7)
C17	0.0243 (7)	0.0125 (6)	0.0235 (6)	−0.0036 (5)	0.0021 (5)	−0.0007 (5)
C18	0.0275 (8)	0.0195 (6)	0.0249 (6)	0.0018 (5)	0.0046 (5)	0.0037 (5)
C19	0.0261 (8)	0.0219 (7)	0.0413 (8)	−0.0044 (6)	0.0085 (6)	0.0039 (6)
C20	0.0412 (9)	0.0235 (7)	0.0275 (7)	−0.0060 (6)	−0.0066 (6)	−0.0038 (6)

### Geometric parameters (Å, °)

**Table:**

O1—C2	1.3683 (15)	C8—H8C	0.9800
O1—C7	1.4264 (15)	C9—H9A	0.9900
O2—C7	1.3907 (16)	C9—H9B	0.9900
O2—C8	1.4272 (16)	C10—H10A	0.9800
O3—C6	1.3754 (16)	C10—H10B	0.9800
O3—C9	1.4210 (15)	C10—H10C	0.9800
O4—C9	1.3894 (16)	C13—C16	1.5109 (19)
O4—C10	1.4272 (18)	C13—C14	1.516 (2)
O5—C11	1.2017 (15)	C13—C15	1.519 (2)
O6—C11	1.3317 (15)	C14—H14A	0.9800
O6—C13	1.4825 (16)	C14—H14B	0.9800
O7—C12	1.2023 (15)	C14—H14C	0.9800
O8—C12	1.3266 (15)	C15—H15A	0.9800
O8—C17	1.4820 (14)	C15—H15B	0.9800
N1—C12	1.4088 (15)	C15—H15C	0.9800
N1—C11	1.4096 (16)	C16—H16A	0.9800
N1—C1	1.4292 (16)	C16—H16B	0.9800
C1—C6	1.3928 (17)	C16—H16C	0.9800
C1—C2	1.3954 (18)	C17—C18	1.5136 (19)
C2—C3	1.3903 (18)	C17—C19	1.5195 (18)
C3—C4	1.3884 (19)	C17—C20	1.5196 (18)
C3—H3	0.9500	C18—H18A	0.9800
C4—C5	1.382 (2)	C18—H18B	0.9800
C4—H4	0.9500	C18—H18C	0.9800
C5—C6	1.3923 (18)	C19—H19A	0.9800
C5—H5	0.9500	C19—H19B	0.9800
C7—H7A	0.9900	C19—H19C	0.9800
C7—H7B	0.9900	C20—H20A	0.9800
C8—H8A	0.9800	C20—H20B	0.9800
C8—H8B	0.9800	C20—H20C	0.9800
			
C2—O1—C7	118.55 (10)	O6—C11—N1	110.18 (10)
C7—O2—C8	112.86 (11)	O7—C12—O8	127.29 (11)
C6—O3—C9	118.13 (10)	O7—C12—N1	121.92 (11)
C9—O4—C10	112.61 (11)	O8—C12—N1	110.71 (10)
C11—O6—C13	120.59 (10)	O6—C13—C16	109.73 (11)
C12—O8—C17	120.00 (9)	O6—C13—C14	110.04 (11)
C12—N1—C11	125.51 (10)	C16—C13—C14	112.79 (13)
C12—N1—C1	116.83 (10)	O6—C13—C15	102.12 (11)
C11—N1—C1	117.61 (10)	C16—C13—C15	110.76 (13)
C6—C1—C2	120.13 (11)	C14—C13—C15	110.89 (13)
C6—C1—N1	120.07 (11)	C13—C14—H14A	109.5
C2—C1—N1	119.80 (11)	C13—C14—H14B	109.5
O1—C2—C3	125.34 (12)	H14A—C14—H14B	109.5
O1—C2—C1	114.49 (11)	C13—C14—H14C	109.5
C3—C2—C1	120.17 (12)	H14A—C14—H14C	109.5
C4—C3—C2	118.65 (12)	H14B—C14—H14C	109.5
C4—C3—H3	120.7	C13—C15—H15A	109.5
C2—C3—H3	120.7	C13—C15—H15B	109.5
C5—C4—C3	122.09 (12)	H15A—C15—H15B	109.5
C5—C4—H4	119.0	C13—C15—H15C	109.5
C3—C4—H4	119.0	H15A—C15—H15C	109.5
C4—C5—C6	118.93 (12)	H15B—C15—H15C	109.5
C4—C5—H5	120.5	C13—C16—H16A	109.5
C6—C5—H5	120.5	C13—C16—H16B	109.5
O3—C6—C5	124.99 (11)	H16A—C16—H16B	109.5
O3—C6—C1	115.00 (11)	C13—C16—H16C	109.5
C5—C6—C1	120.01 (12)	H16A—C16—H16C	109.5
O2—C7—O1	113.19 (10)	H16B—C16—H16C	109.5
O2—C7—H7A	108.9	O8—C17—C18	110.43 (10)
O1—C7—H7A	108.9	O8—C17—C19	101.58 (10)
O2—C7—H7B	108.9	C18—C17—C19	110.37 (11)
O1—C7—H7B	108.9	O8—C17—C20	109.33 (10)
H7A—C7—H7B	107.8	C18—C17—C20	113.10 (11)
O2—C8—H8A	109.5	C19—C17—C20	111.43 (12)
O2—C8—H8B	109.5	C17—C18—H18A	109.5
H8A—C8—H8B	109.5	C17—C18—H18B	109.5
O2—C8—H8C	109.5	H18A—C18—H18B	109.5
H8A—C8—H8C	109.5	C17—C18—H18C	109.5
H8B—C8—H8C	109.5	H18A—C18—H18C	109.5
O4—C9—O3	113.22 (10)	H18B—C18—H18C	109.5
O4—C9—H9A	108.9	C17—C19—H19A	109.5
O3—C9—H9A	108.9	C17—C19—H19B	109.5
O4—C9—H9B	108.9	H19A—C19—H19B	109.5
O3—C9—H9B	108.9	C17—C19—H19C	109.5
H9A—C9—H9B	107.7	H19A—C19—H19C	109.5
O4—C10—H10A	109.5	H19B—C19—H19C	109.5
O4—C10—H10B	109.5	C17—C20—H20A	109.5
H10A—C10—H10B	109.5	C17—C20—H20B	109.5
O4—C10—H10C	109.5	H20A—C20—H20B	109.5
H10A—C10—H10C	109.5	C17—C20—H20C	109.5
H10B—C10—H10C	109.5	H20A—C20—H20C	109.5
O5—C11—O6	127.26 (12)	H20B—C20—H20C	109.5
O5—C11—N1	122.45 (11)		
			
C2—C1—N1—C12	57.65 (15)	C6—C1—N1—C11	59.68 (15)
